# Overexpression of human kynurenine-3-monooxygenase protects against 3-hydroxykynurenine-mediated apoptosis through bidirectional nonlinear feedback

**DOI:** 10.1038/cddis.2016.87

**Published:** 2016-04-14

**Authors:** K Wilson, M Auer, M Binnie, X Zheng, N T Pham, J P Iredale, S P Webster, D J Mole

**Affiliations:** 1 Drug Discovery Core, University/BHF Centre for Cardiovascular Science, Queen's Medical Research Institute, The University of Edinburgh, 47 Little France Crescent, Edinburgh, UK; 2 School of Biological Sciences and School of Biomedical Sciences, The University of Edinburgh, C.H. Waddington Building, King's Buildings, Max Born Crescent, Edinburgh, UK; 3 MRC Centre for Inflammation Research, Queen's Medical Research Institute, The University of Edinburgh, 47 Little France Crescent, Edinburgh, UK

## Abstract

Kynurenine 3-monooxygenase (KMO) is a critical regulator of inflammation. The preferred KMO substrate, kynurenine, is converted to 3-hydroxykynurenine (3HK), and this product exhibits cytotoxicity through mechanisms that culminate in apoptosis. Here, we report that overexpression of human KMO with orthotopic localisation to mitochondria creates a metabolic environment during which the cell exhibits increased tolerance for exogenous 3HK-mediated cellular injury. Using the selective KMO inhibitor Ro61-8048, we show that KMO enzyme function is essential for cellular protection. Pan-caspase inhibition with Z-VAD-FMK confirmed apoptosis as the mode of cell death. By defining expression of pathway components upstream and downstream of KMO, we observed alterations in other key kynurenine pathway components, particularly tryptophan-2,3-dioxygenase upregulation, through bidirectional nonlinear feedback. KMO overexpression also increased expression of inducible nitric oxide synthase (iNOS). These changes in gene expression are functionally relevant, because siRNA knockdown of the pathway components kynureninase and quinolinate phosphoribosyl transferase caused cells to revert to a state of susceptibility to 3HK-mediated apoptosis. In summary, KMO overexpression, and importantly KMO activity, have metabolic repercussions that fundamentally affect resistance to cell stress.

The kynurenine pathway is the main route of tryptophan (TRP) metabolism in mammals (Figure 1). Kynurenine 3-monooxygenase (KMO) is a flavoprotein hydroxylase enzyme that catalyses the conversion of kynurenine (KYN) to 3-hydroxykynurenine (3HK) in the kynurenine pathway. KMO is an important therapeutic target for multiple organ dysfunction, particularly that triggered by acute pancreatitis and the systemic inflammatory response,^1, 2^ and for Huntington's disease.^3^ KMO also has a significant role in the immune adaptive response.^4^ TRP is converted to KYN by tryptophan-2,3-dioxygenase (TDO) and indoleamine-2,3-dioxygenases (IDOs), following which KYN has several potential fates. The majority of KYN is metabolised by KMO to 3HK. KYN is also a substrate for kynurenine aminotransferase 1 and 2 (KAT1 and KAT2) to form kynurenic acid (KYNA). KYNA is sedative and neuroprotective, acting at GABA (*γ*-aminobutyric acid) receptors. Hypothetically, KYN may also be directly converted to 3-hydroxyanthranilic acid (3HAA) but most 3HAA is produced by the oxidation of 3HK by 3-hydroxyanthranilic acid oxidase (3HAO). 3HAA is subsequently metabolised to quinolinic acid (QUIN), which is injurious to cells, especially neurons, via a mechanism of NMDA-mediated excitotoxicity.^5^ The kynurenine pathway culminates in the production of nicotinamide adenine dinucleotide (NAD).^4^

Increased kynurenine metabolite levels have been implicated in many disease states, particularly in neurodegenerative and inflammatory disorders. In particular, the KMO product, 3HK, is a free radical generator that exhibits toxicity to cells through reactive oxygen species generation, crosslinking of proteins and mitochondrial respiratory chain inhibition.^6^ 3HK has recently been identified in a metabolomic screen of brain tissue extracts in Parkinson's disease.^7^ Therefore, because systemic inflammatory disease states are associated with increased flux through the kynurenine pathway^1, 8^ and because we and others have previously observed 3HK-mediated cytotoxicity *in vitro* we wanted to investigate whether increased expression of KMO in a mammalian system affects the cell death response to 3HK, and, if so, to explore the potential underlying mechanisms.

To address this question we overexpressed KMO in HEK293 cells and imaged the subcellular localisation of overexpressed KMO. The cell death response to exogenous 3HK was then evaluated by three separate measures of cytotoxicity and subsequently confirmed by direct visualisation using time-lapse confocal fluorescence microscopy of cells overexpressing a fluorescent KMO fusion protein. To define whether altered sensitivity to 3HK-mediated cell death was dependent on KMO activity we used the potent KMO inhibitor Ro61-8048. We measured the effect of KMO overexpression on upstream and downstream kynurenine pathway enzyme expression and evaluated the functional relevance of gene silencing using siRNA knockdown of specific pathway components. Lastly, we propose a mechanism to explain these observations as our experiments show that KMO-overexpressing cells undergo bidirectional adaptation via alteration of kynurenine pathway homoeostasis.

## Results

### Human KMO stably expressed in HEK293 cells is enzymatically active and co-localises to the mitochondria

**Figure 2:**
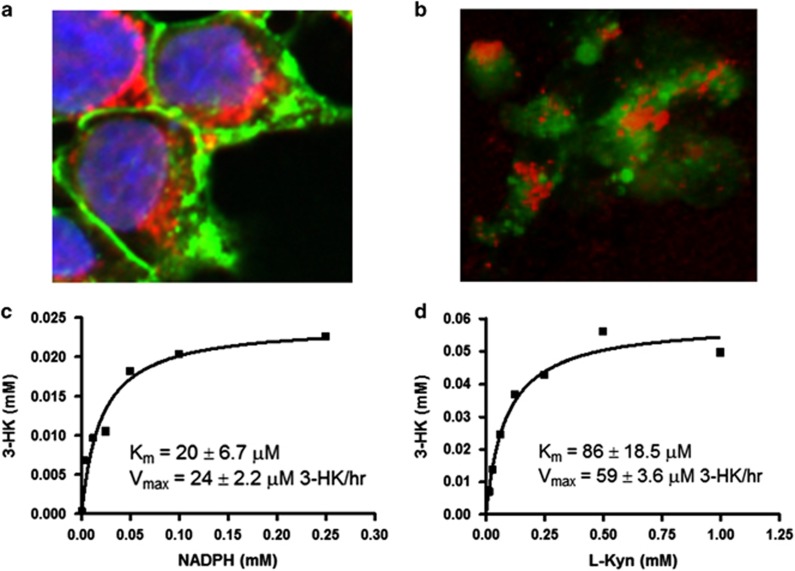
Expression of active mitochondrial localised KMO. (**a**) Cellular staining image indicating mitochondrial localisation of KMO in HEK-KMO(V5-6His) cells obtained using the Opera HCS system with a × 40 water immersion objective (NA 0.9). Antibody-labelled KMO was detected using the 640 nm laser (2000 *μ*W, 40 ms exposure time, emission filter 690/70), nuclear staining was detected using the UV light source (365 nm excitation, 40 ms, emission filter 450/50) and the 488 nm laser (1250 *μ*W, 280 ms, emission filter 520/35) was used to detect the cell membrane stain. The white scale bar corresponds to 10 *μ*m. (**b**) 3D image of KMO-expressing cells obtained using the Leica SP5C spectral confocal laser scanning microscope. The argon (488 nm) laser was used for detection of mitochondria and the 633 nm laser for detection of KMO confirming co-localisation. The white scale bar corresponds to 10 *μ*m. Steady-state kinetics are shown for KMO at 37 °C, pH 7.0. Starting concentrations of (**c**) NADPH and (**d**) l-kynurenine are plotted versus 3HK produced and data fitted to the Michaelis–Menten equation (*Y*=Bmax*X/(*K*_d_+*X*) using GraphPad Prism4 software

KMO detected with anti-V5-Dylight650 antibody was localised in the cytoplasm in the perinuclear region of the cell consistent with the distribution of mitochondria in cells^9^ (Figure 2a). Three-dimensional analysis of HEK293-E2-Crimson-KMO cellular staining images verified co-localisation of KMO to the mitochondria (stained with MitoGreen; PromoKine, Heidelberg, Germany) (Figure 2b) with a significant Pearson correlation coefficient of 44.2%. This correlation result indicates a strong positive relationship between the localisation of the mitochondria and KMO in these cells.

Full-length KMO(V5-6His) demonstrated enzymatic activity in the cell lysate with a *K*_m_ for NADPH of 20±6.7 *μ*M and a *K*_m_ for l-kynurenine of 86±18.5 *μ*M (Figure 2c and d).

### KMO-overexpressing cells are protected from 3HK-mediated toxicity as a function of KMO activity

**Figure 3:**
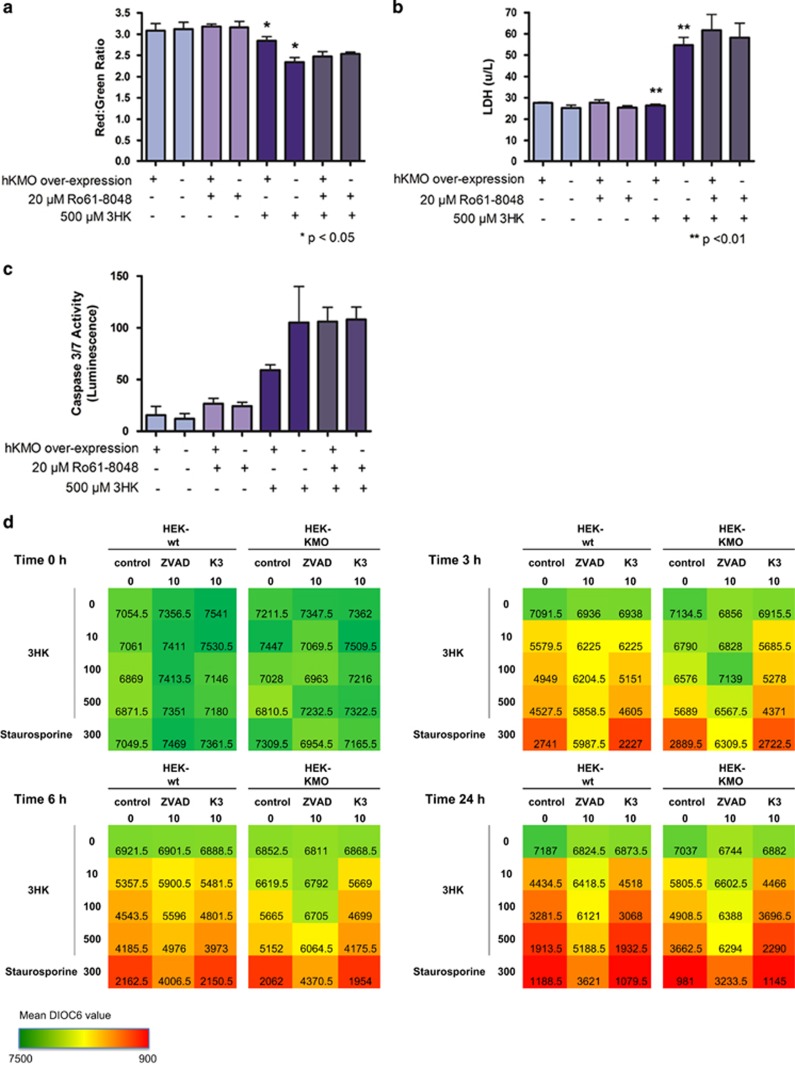
KMO protects against 3HK-induced cell death. Graphs showing (**a**) JC-1 readout, *n*=6 for each cell type and conditions, red:green ratio is significantly decreased (*P*=0.0498) in wild-type cells incubated with 3HK compared with KMO cells, (**b**) LDH assay readout, *n*=6 for each cell type and conditions, LDH release is significantly increased (*P*=0.0016) in wild-type cells incubated with 3HK compared with KMO cells, (**c**) caspase 3/7 activity readout, *n*=6 for each cell type and conditions, caspase activity is increased in wild-type cells incubated with 3HK compared with KMO cells. The S.E.M. is indicated by error bars on all graphs. Data are representative of three independent experiments for each of these assays. (**d**) Heat maps indicating DiOC6 staining in KMO cells verses wild-type cells with a 3HK-dose and time response in the presence and absence of Z-VAD-FMK, data are mean values of *n*=2 for each cell type and conditions from one independent experiment

Time-lapse bright-field microscopy of stably transfected HEK293-KMO(V5-6His) cells did not show any changes in baseline cell death when unchallenged. However, and in keeping with our previous observations of 3HK-mediated cytotoxicity,^1^ addition of 500 *μ*M 3HK caused cell death in wild-type HEK293 cells (Figure 3a–d and Supplementary Video 1). Interestingly, overexpression of KMO protected cells against 3HK-mediated cytotoxicity when quantified by measuring LDH release into the cell culture media (Figure 3b). Cell death was preceded by a loss of mitochondrial transmembrane potential demonstrated by DiOC6 staining (Figure 3d) and measured using the intravital dye JC-1 (Figure 3a). Again, protection against 3HK-mediated mitochondrial injury was conferred by overexpression of KMO. Furthermore, caspase activity was significantly increased in cells exposed to 3HK (Figure 3c), confirming activation of canonical apoptosis pathways, but this activation was much less pronounced in KMO-overexpressing cells compared with non-transfected HEK293 control cells. Moreover the cytoprotective effect was abrogated by addition of the potent KMO inhibitor Ro61-8048^10^ (Figure 3a–d), thus establishing that overexpressed KMO must be functional to be protective. Addition of the pan-caspase inhibitor Z-VAD-FMK decreased the disruption of mitochondrial transmembrane potential measured by DiOC6 further validating the mode of 3HK-induced cell death as apoptosis (Figure 3d).

**Figure 4:**
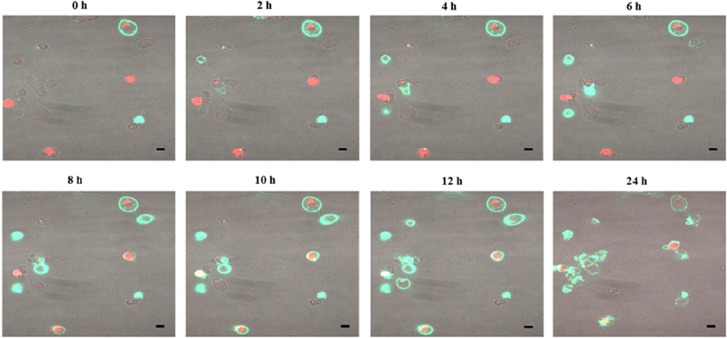
Time-lapse confocal microscopy images of HEK293 cells transiently transfected with 10His-E2-Crimson-KMO (red) incubated with 500 *μ*M 3HK in the presence of Annexin-V-Fluos (green) showing low or non-KMO-expressing cells undergoing apoptosis more rapidly than high-expressing cells. The black scale bar corresponds to 10 *μ*m

In order to prove that (i) the protective effect of overexpressing KMO was proportional to the amount of KMO activity and, (ii) that protection was conferred at the level of the individual cell rather than on an ambient or bystander effect in an entire cell culture, HEK293 cells were transiently transfected with the 10His-E2-Crimson-KMO fusion protein and visualised by confocal time-lapse microscopy. Transient transfection resulted in a range of E2-Crimson-KMO overexpression in cells in a single-culture vessel, allowing the visual comparison of E2-Crimson fluorescence intensity (and therefore KMO activity) between cells to act as an internal control within one culture vessel. Cells variably expressing E2-Crimson-KMO were incubated with 500 *μ*M 3HK and studied under time-lapse conditions. Cells with lower E2-Crimson fluorescence intensity, and therefore lower KMO activity, were markedly more susceptible to 3HK-mediated cytotoxicity, undergoing apoptosis sooner than cells in the same culture which had high overexpression of E2-Crimson-KMO (Figure 4 and Supplementary Video 1).

Taken together, these data show that overexpression of KMO protects against 3HK-mediated cell death and that KMO enzyme activity is both necessary and proportionate to that protection.

### Overexpression of KMO alters endogenous kynurenine pathway homoeostasis

**Figure 5:**
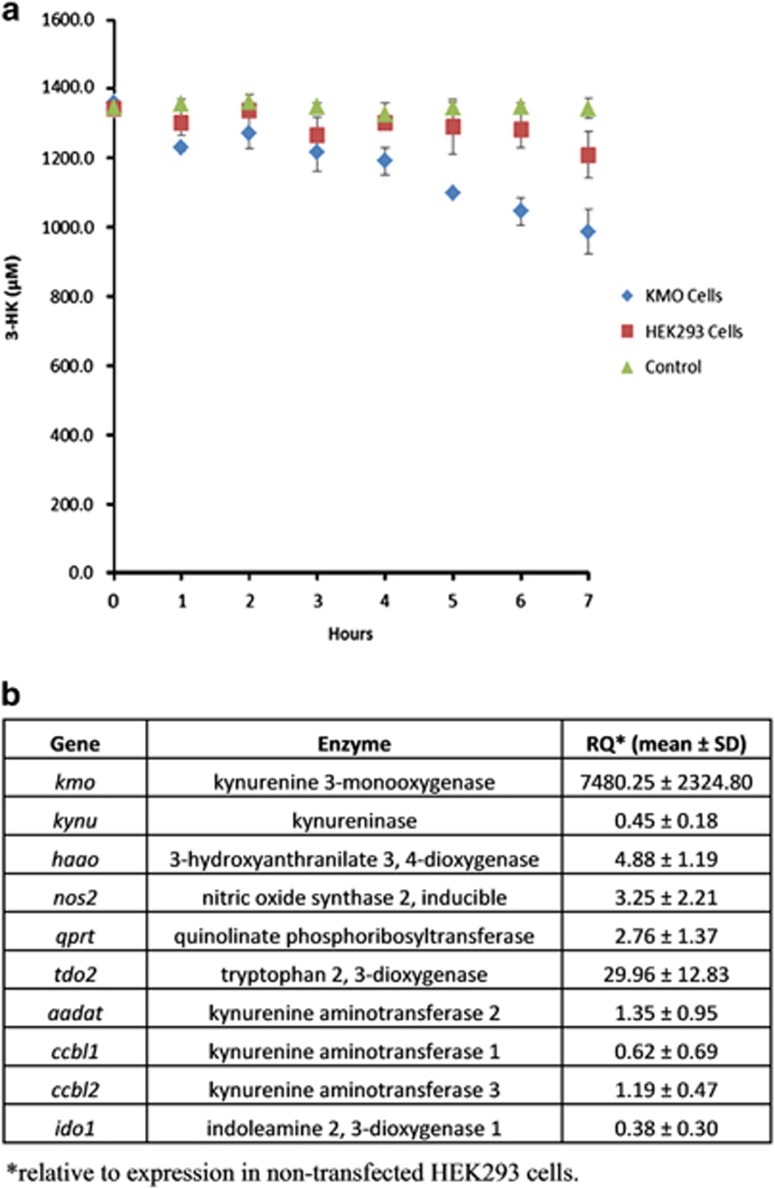
Quantification of exogenous 3HK and KMO mRNA levels. (**a**) Plot showing 3HK concentration remaining in each cell and control incubation plotted against incubation time, *n*=4, the S.E.M. is indicated by error bars on the plot, data are representative of two independent experiments. (**b**) Table showing relative quantification of mRNA levels corresponding to each kynurenine pathway enzyme in HEK-KMO and HEK293 cells as determined by RT-qPCR

One possible explanation for the observation that overexpression of KMO confers resistance to 3HK-mediated cell death is that cells which overexpress KMO are capable of rapid elimination of 3HK from the ambient medium. Therefore, we measured the rate of disappearance of 3HK from the medium in KMO-overexpressing cells compared with controls. When incubated with HEK-KMO(V5-6His) cells, 3HK concentration in the medium reduced in a time-dependent manner at a much faster rate than in wild-type HEK293 cell cultures, indicating more rapid clearance, sequestration or metabolism of 3HK (Figure 5a).

To investigate further whether increased flux through the kynurenine pathway observed in HEK-KMO(V5-6His) cells resulted from a global increase in kynurenine pathway enzyme expression, we measured mRNA expression of upstream and downstream components in HEK-KMO(V5-6His) cells compared with wild-type control cells. KMO-overexpressing cells showed significantly increased TDO mRNA expression (30-fold). HAAO (3-hydroxyanthranilate 3, 4-dioxygenase) and QPRT (quinolinate phosphoribosyl transferase) mRNA expression were also elevated (Figure 5b). Interestingly, although IDO1 is expressed at low levels in HEK cells, no increase in expression accompanied increased flux through the pathway, and IDO1 expression did not increase in line with increased TDO expression. KYNU (kynureninase), KAT1 (kynurenine amino transferase-1), KAT2 and KAT3 expression were not significantly altered.

### Overexpression of KMO increases iNOS expression

It is known that 3HK acts as a free radical donor, therefore the increased substrate flux through KMO and resultant increase in 3HK production was investigated as a potential oxidative stressor. Inducible nitric oxide synthase-2 (iNOS) mRNA expression was measured as an indicator of oxidative stress. iNOS expression was increased 3.25-fold in KMO-overexpressing cells compared with controls (Figure 5b).

### siRNA knockdown of KYNU and QPRT in KMO-overexpressing cells restores susceptibility to 3HK-mediated cell death

**Figure 6:**
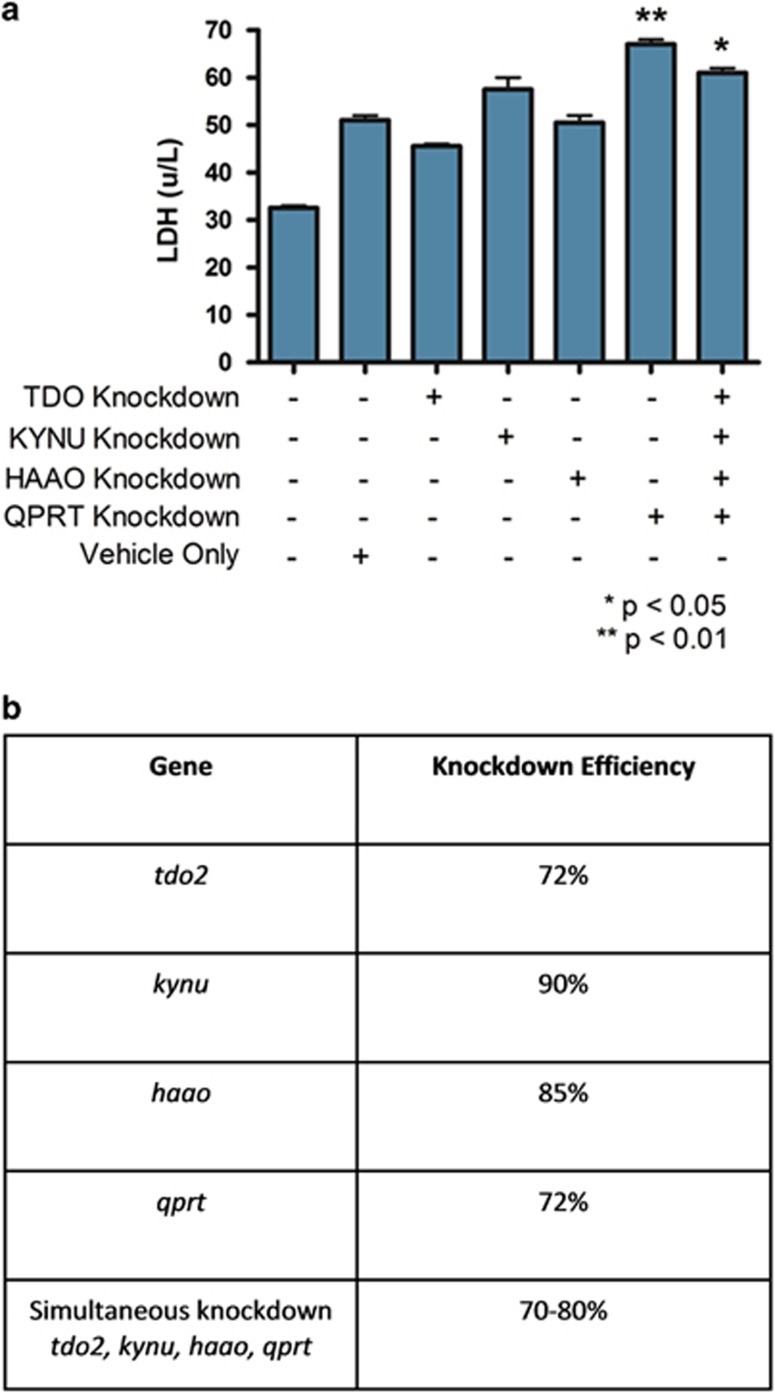
siRNA knockdown of the kynunenine pathway. (**a**) Graph showing cell death response indicated by LDH assay, *n*=2 for each knockdown conditions, the S.E.M. is indicated by error bars on the graph, data are from one independent experiment, knockdown of QPRT causes a significant increase (*P*=0.0077) in cell death as does knockdown of all four genes (*P*=0.0194). (**b**) Table showing the averaged knockdown efficiency for each gene in the cells

To determine whether the alterations in pathway enzyme gene expression were functionally relevant to the observed protection from 3HK-mediated cytotoxicity, we used siRNA knockdown, singly and in combination, for the kynurenine pathway components listed in Figure 6. Knockdown efficiencies for each gene targeted are shown in Figure 6b. Vehicle only control incubations (containing Lipofectamine RNAiMAX) caused a small increase in susceptibility to 3HK-mediated cytotoxicity. However, a significant further increase in susceptibility to 3HK was seen after siRNA knockdown of kynureninase (KYNU) and QPRT. siRNA knockdown of TDO and HAAO showed no difference in 3HK-mediated cytotoxicity compared with transfection reagent alone. In keeping with these observations, knockdown of all four pathway components simultaneously confirmed the phenotypic effect of KYNU and QPRT knockdown alone, but did not show any additive effect. Together, these data confirm that the alterations in kynurenine pathway enzyme mRNA expression are a functional component of the mechanism by which KMO overexpression affords protection against 3HK-mediated cytotoxicity.

## Discussion

KMO is a critical component of the kynurenine pathway of tryptophan metabolism and an important regulator of inflammation. In this series of experiments, we demonstrate that overexpression of human KMO with orthotopic localisation to mitochondria creates a metabolic environment, in which the cell is resistant to exogenous 3HK-mediated cellular injury. Using a potent KMO inhibitor we show that protection depends on KMO enzyme function and further substantiate this concept by directly visualising 3HK-mediated death in cells with low or no expression of KMO and increased tolerance to exogenous 3HK in high KMO-expressing cells in a single-culture system. Therefore, we have shown that the protective effect of KMO overexpression is directly proportional to the amount of active KMO present in any individual cell. KMO overexpression and protection was accompanied by an increased elimination of 3HK from ambient culture medium. Cells with increased KMO activity also showed transcriptional upregulation of the upstream enzyme TDO and the downstream kynurenine pathway components HAAO and QPRT. These changes, in particular KYNU and QPRT upregulation, were shown to be more than associative observations because siRNA knockdown of KYNU and QPRT restored susceptibility to 3HK-mediated cell death in HEK-KMO cells.

KMO is localised to the outer mitochondrial membrane due to the presence of a large membrane targeting domain at its C-terminus.^11^ The tissue distribution of KMO is largely in liver and kidney, but lower expression is found in brain, especially microglial cells and macrophages. Artificial overexpression of human KMO protein has been reported in the following cell lines: HEK293,^12^ COS-1 (ref. 13) and CHO cells.^14^ Winkler *et al.*^14^ reported localisation of exogenous human KMO to the mitochondria in CHO cells using immunohistochemistry. Congruent with this, cell-staining experiments reported herein show detection of KMO staining around the nucleus of the cell (Figure 2a). This is consistent with the perinuclear mitochondrial distribution in the cell^9^ and indicates that recombinant expression of human KMO in these cells mimics localisation observed in natural cells. 3D imaging analysis confirmed co-localisation of KMO to the mitochondria with a significant Pearson correlation coefficient of 44.2%. KMO demonstrated good enzymatic activity (Figure 2c and d) when prepared in a cell lysate that was comparable to previously reported kinetic characterisation of the human enzyme.^13^

3HK is an oxidative stress generator that releases free hydroxyl radicals inducing cellular apoptosis.^15^ 3HK sensitivity varies between tissue/cell types and differential uptake of 3HK by cells is believed to be responsible.^16^ Wei *et al.*^16^ tested 3HK toxicity against three neuronal cell types and found all three to be sensitive to high pharmacological doses of 3HK with at least 90% cell loss at a concentration of 500 *μ*M. KMO-overexpressing cells demonstrated reduced cell death at this concentration than wild-type HEK293 cells (Figure 3). In order to define the requirement for KMO enzyme activity to confer protection, Ro61-8048, a known KMO inhibitor,^10^ was used for the duration of the 3HK incubation to define KMO activity dependence in these cells. KMO blockade restored sensitivity to 3HK-mediated cell death to a level similar to that of the wild-type cells. Three measures of cytotoxicity were used, namely membrane depolarisation, plasma membrane rupture and initiation of apoptosis were used to validate this result (Figure 3a). Detection of caspases 3 and 7 activity in cell lysates and a reduction in cell death response following pan-caspase inhibition confirms that the mechanism of 3HK-mediated cell death is apoptosis, as previously indicated in the literature.^16, 17^

Exogenous 3HK concentration decreased steadily over time in KMO-overexpressing cells but not in the HEK293 wild-type incubation and in the cell-free controls, indicating increased metabolism or clearance of 3HK. This adaptation offers a potential explanation for altered sensitivity to 3HK-mediated cell death in KMO-overexpressing cells.

Metabolism of 3HK necessitates expression of the enzymes downstream of KMO in the kynurenine pathway. The mRNA levels corresponding to the genes for the KP enzymes were quantified for each cell type and used as a surrogate indicator of protein expression. mRNA levels corresponding to KMO were 7000 times higher in the transfected cells than in the wild-type cells. HEK293 cells express KMO endogenously and the other kynurenine pathway enzymes but KMO activity was not detectable in these cells (data not shown), correlating with the observations of Alberati-Giani *et al.*^12^ Several kynurenine pathway enzymes demonstrated upregulation in the KMO cells, in particular, the gene corresponding to TDO. TDO catalyses the first and rate limiting step in tryptophan metabolism. TDO upregulation in KMO-overexpressing cells may imply the existence of a feedback mechanism to enable homoeostasis of kynurenine concentrations in the cell. Overexpression of KMO results in increased turnover of kynurenine to 3HK, it is possible that TDO is upregulated to maintain kynurenine levels by production of the kynurenine precursor metabolite N-formylkynurenine. 3-HAAO and QPRT also demonstrated upregulation. This may contribute to the decreasing 3HK concentration as these enzymes occur downstream of KMO in the pathway. A further possible explanation is that metabolism of 3HK by upregulated HAAO and QPRT may increase tolerance to 3HK as clearance of 3HK will be more efficient in KMO-overexpressing cells. This argument is rational and precedented by observations in several other biological systems that are able to adapt in response to external stimuli for the maintenance of physiological homoeostasis.^18^ Integral feedback control is an important element in ensuring homoeostasis in a variable or uncertain environment when the surrounding parameters are imperfect.^19^ Specifically, in our engineered cell line system, we have overexpressed KMO protein by 7000-fold. It is therefore expected that cellular kynurenine levels will be depleted and 3HK levels elevated due to increased KMO activity, although this was not directly measured. We did show that the upstream enzyme TDO and downstream enzymes HAAO and QPRT are upregulated in these cells, and can hypothesise that these changes are the result of integral feedback control in this cell system in response to altered kynurenine and 3HK levels. Increased kynurenine production stimulated by TDO and increased 3HK metabolism by HAAO and QPRT may facilitate maintenance of a steady-state environment despite KMO overexpression. Upregulation of the downstream enzymes may also contribute to reduced 3HK mediated cell death by 3HK metabolism.

The interactome of KMO is not yet established. Of interest in our paper is the upregulation of iNOS2 mRNA levels in KMO-overexpressing cells. Elevated levels of the reactive nitric oxide free radical NO produced by iNOS2 are associated with cellular stress.^20^ KMO is pro-apoptotic due to its production of free radical generating 3HK.^17^ Whilst the results described here support reduced 3HK-mediated cell death in KMO-expressing cells, increased production of 3HK may induce cellular stress indicated by upregulated iNOS2. In addition, production of neurotoxic quinilonic acid may contribute to this cellular stress as a result of upregulated HAAO expression.

It was important to determine that the altered cell death response resulted from KMO expression and activity rather than upregulation of other kynurenine pathway enzymes. TDO and three enzymes downstream of KMO were downregulated by siRNA knockdown before incubation with 3HK. Knockdown of TDO and HAAO showed no effect on the cell death response to 3HK. mRNA levels corresponding to KYNU expression were not upregulated following overexpression of KMO in HEK293 cells indicating that 3HK can be sufficiently metabolised by KYNU and the KAT enzymes at baseline levels in these cells. However, knockdown of KYNU gene expression (the enzyme directly downstream of KMO) resulted in increased 3HK-mediated cell death. Since KYNU is placed in the main and primary branch of the kynurenine pathway, knockdown of this enzyme will impact upon 3HK metabolism enabling accumulation of 3HK. Such alterations to 3HK levels are consistent with the observation of increased cell death. This indicates that protection against 3HK-mediated cytotoxicity is lost when metabolism of 3HK is prevented in these cells. Knockdown of QPRT also resulted in increased 3HK-mediated cell death; however, it is likely that a separate mechanism is contributory to this finding. Although QPRT metabolises neurotoxic quinilonic acid to produce NAD as the final step of the kynurenine pathway and quinilonic acid accumulation in the cells may contribute to the increased cell death response, QPRT is also responsible for production of NAD in the pathways of alanine, aspartate and glutamate metabolism. Therefore, QPRT knockdown will not only impact on the kynurenine pathway but also on several other precursor pathways of NAD synthesis. Prevention of the biosynthesis of this essential cellular co-factor would result in cell death, as observed in our experiments.

We have shown that functional KMO overexpression is responsible for reduced sensitivity to 3HK-mediated cell death. Furthermore, we have shown that functional reversal of this overexpression with KMO inhibition using a small molecule inhibitor and prevention of 3HK metabolism by KYNU siRNA knockdown results in restoration of a normal 3HK-mediated cell death response. We can therefore conclude that KMO-overexpressing cells exhibit bidirectional adaptation through altered kynurenine pathway enzyme concentrations upstream and downstream of overexpressed KMO. These adaptations result in the maintenance of kynurenine and 3HK at an appropriate level in the cell in an example of robust integral feedback control following a nonlinear form. Protection from 3HK toxicity appears to be an important and interesting secondary effect of altered kynurenine pathway homoeostasis.

Overexpression of KMO in HEK293 cells has allowed the observation of several KMO-mediated effects and the characterisation of KMO at a cellular level in an engineered expression system. Whether these KMO-mediated effects are physiologically relevant in a naturally-regulated or disease-regulated cell system is now important to determine. In summary, human KMO overexpression, and importantly activity, has metabolic repercussions that fundamentally affect resistance to 3HK-mediated cell stress.

## Materials and Methods

### Cloning

#### KMO(V5-6His)

Full-length human KMO (GenBank Accession No. NM_003679) (Cys 452 variant) was amplified from an image clone by Taq polymerase-amplified PCR. A KOZAK sequence for translational initiation was incorporated by the forward PCR primer. The native stop codon was removed by the reverse PCR primer. The gene was ligated into the pcDNA5/FRT/V5/HisTOPO vector (Life Technologies, Renfrew, UK) by TOPO cloning reaction to facilitate expression by the Flp-In system. The vector C-terminal V5 and 6 histidine tags were incorporated in the protein construct.

#### 10His-E2-Crimson-KMO

The fusion construct gene 10His-E2-Crimson-KMO was synthesised by Genscript and provided in vector pUC57. This construct incorporated a 10 × polyhistidine tag (CATCATCATCACCATCATCATCATCATCAC), far-red fluorescent protein E2-crimson (sequence obtained from Clontech, CA, USA) followed by an enterokinase site (GATGACGATGACAAG) and full-length human KMO (GenBank Accession No. NM_003679, Cys 452 variant). A KOZAK sequence was incorporated before the start codon for initiation of translation in mammalian cells. The gene was ligated in to vector pcDNA3.1 (Life Technologies).

### Expression

#### KMO(V5-6His)

HEK293 (Flp-In-293 which express lacZ-Zeocin, Life Technologies) cells were stably co-transfected with 9 *μ*g of pcDNA5/FRT/V5/HisTOPO DNA and 18 *μ*g of pog44 plasmid. Pog44 expresses Flp recombinase protein; co-transfection of pog44 with the gene of interest allows targeted integration into the mammalian cell genome within a transcriptionally active region. Transfection was carried out using Lipofectamine 2000 (Life Technologies) in OPTI-MEM medium (Lonza, Slough, UK). Cells were selected for two weeks using hygromycin B (Sigma Aldrich, Dorset, UK) at 100 *μ*g/ml to select for the hygromycin resistance gene contained within the pcDNA5 vector before positive colonies were isolated and cultured.

#### 10His-E2-Crimson-KMO

HEK293 cells were transiently transfected with pcDNA3.1-10His-E2-Crimson-KMO plasmid DNA. Transfection was carried out using Lipofectamine 2000 in OPTI-MEM medium. Cells were used for staining and time-lapse experiments 24–48 h post transfection.

#### KMO activity assay: kinetic characterisation

Cell lysates were analysed for KMO enzymatic activity by measuring the amount of KYN converted to 3HK detected using liquid chromatography-mass spectrometry (LC-MS/MS). Briefly, 200 *μ*g total protein was incubated with 4 mM MgCl_2_, 1 mM NADPH and 200 *μ*M l-kynurenine in 20 mM HEPES, pH 7 for 2 h at 37 °C with gentle shaking at 250 r.p.m. in a total assay volume of 100 *μ*l. Samples were added to 500 *μ*l acetonitrile (containing 25 *μ*g/ml of internal standard, d5-TRP) to terminate activity, followed by centrifugation at 4000 r.p.m. for 20 min to pellet the precipitate. The supernatant containing KYN and 3HK was removed, dried under nitrogen, and the residue re-suspended in 30 : 70 methanol:water with 0.1% formic acid ready for LC-MS/MS analysis.

For the determination of steady-state kinetic parameters for substrate l-kynurenine, NADPH was added at a concentration of 2 mM with glucose-6 phosphate (3 mM) and glucose-6 phosphate dehydrogenase (1 unit of enzyme per assay) to maintain the NADPH concentration. l-kynurenine was added at concentrations of 2, 1, 0.5, 0.25, 0.125, 0.062, 0.031 and 0.015 mM. For co-factor NADPH dependence of the enzymatic activity, l-kynurenine was added in excess at a concentration of 500 *μ*M. NADPH was added at a concentration of 100, 50, 25, 12.5, 6.2, 3.1, 1.5 and 0.75 *μ*M. Assays were performed in duplicate.

LC-MS analysis was carried out using the TSQ Quantum Discovery triple quadrupole mass spectrometer (Thermo Fisher Scientific, Hemel Hempstead, UK) using a pentafluorophenyl (PFP) fused pore column (Waters, Hertfordshire, UK), at 40 °C. The injection volume was 10 *μ*l and the flow rate was 500 *μ*l/min. The method had a run time of 4 min and d5 tryptophan was used as an internal standard. Qualifier and quantifier peaks were identified for 3HK and for d5 Tryptophan. Data was acquired and processed using Xcalibur 1.4 and LC Quan 2.0 SP1 (Thermo Scientific, Leicestershire, UK) software packages.

### Staining cells for immunofluorescence

HEK-KMO(V5-6His) cells were stained in transparent 96-well plates (Whatman, GE Healthcare, Buckinghamshire, UK). Wheat germ agglutinin Alexa Fluor 488 conjugate (Life Technologies) was used to stain the plasma membrane of the cells. Cells were then fixed by ten minute incubation in 3.7% paraformaldehyde diluted in PBS^+^. Following fixation, cells were permeabilised (0.1% Triton-X100 in PBS) and blocked (3% BSA in PBS) before staining for KMO using anti-V5 antibody (Life Technologies) chemically labelled with DyLight650 (Pierce, Thermo Scientific, Leicestershire, UK). Finally, cells were incubated with DAPI (Biolegend, CA, USA) to allow nuclear staining.

For z stack imaging, HEK-10His-E2-Crimson-KMO cells were fixed and permeabilised as above and stained for mitochondria using MitoGreen (PromoKine).

### Cellular imaging

Cellular staining images were obtained using the Opera High Content Screening (HCS) system (Perkin Elmer, Coventry, UK). Anti-V5-DyLight650-labelled KMO was detected using the 640 nm laser (2000 *μ*W, 40 ms exposure time, and 690/70 emission filter), DAPI (nuclear) staining was detected using the UV light source (365 nm excitation, 40 ms exposure time, 450/50 emission filter) and the 488 nm laser (1250 *μ*W, 280 ms exposure time, and 520/35 emission filter) was used to detect the cell membrane stain (wheat germ agglutinin-Alexa488 conjugate). A 40x water immersion objective (NA 0.9) was used for all imaging.

### Z stack imaging

A Leica SP5C spectral confocal laser scanning microscope (Leica Microsystems, Milton Keynes, UK) was used to obtain high-resolution Z stack images for 3D imaging analysis. Co-localisation images were obtained using a × 40 objective Plan-Apochromat oil immersion lens and the Argon (488 nm) and 633 nm lasers at Nyquist sampling rate. Data was collected using Leica Application Suite Advanced Fluorescence (LAS AF) software. The images were analysed using FACSDiva software (BD Bioscience, Oxford, UK) to determine the Pearson coefficient for co-localisation.

### JC-1 assay

Non-transfected HEK293 cells and HEK-KMO(V5-6His) cells were plated out separately into 96-well plates at 5 × 10^4^ cells per well and incubated overnight at 37 °C, 5% CO_2_, 95% O_2_ in DMEM with 10% FBS, 1% l-glutamine, 1% penicillin-streptomycin. The next day, tissue culture medium was replaced with medium containing 500 *μ*M 3HK and 20 *μ*M Ro61-8048 (ref. 10) (Roche, West Sussex, UK), a potent inhibitor of KMO, on both cell types in triplicate and incubated overnight for 24 h. Controls consisted of both cell types incubated with 500 *μ*M 3HK only, 20 *μ*M Ro-618048 only and medium only. After incubation, overnight medium was replaced with medium containing 10 *μ*g/ml JC-1 dye (Life Technologies) and cells incubated for 30 min in the dark. JC-1 is a lipophilic cationic dye which is used as an indicator of cell health. The dye was replaced with fresh medium before reading fluorescence intensity on the TECAN infinite M1000 plate reader (Tecan, Reading, UK). The plate was read at excitation 535 nm, emission 590 nm to determine the red measurement and at excitation 485 nm and emission 530 nm to determine the green measurement. Measurements were averaged and the red:green ratio was determined for each sample. JC-1 dye enters mitochondria and its colour changes to correspond with membrane potential, changes in membrane potential are represented by the red:green ratio (red—healthy cells, green—unhealthy cells).

### LDH assay

Non-transfected HEK293 cells and HEK-KMO(V5-His) cells were plated out and incubated overnight as detailed above. The following day, the cells were subjected to the same 3HK and Ro61-8048 conditions as described above for 24 h. After incubation, cell supernatants were sampled and LDH activity was measured. LDH catalyses the reaction between Pyruvate and NADH_2_ resulting in the formation of lactate and NAD. Enzyme activity was obtained by measuring the rate of decrease in absorbance at 340 nm due to the oxidation of NADH_2_ using a commercial kit (Alpha Laboratories Ltd., Eastleigh, UK) adapted for use on a Cobas Fara centrifugal analyser (Roche Diagnostics Ltd). LDH assay is an indicator of plasma membrane integrity as lactate dehydrogenase is only released and detectable in the cellular supernatant when cell membranes rupture.

### Caspase 3 and 7 activity assay

Non-transfected HEK293 cells and HEK-KMO(V5-His) cells were plated out and incubated overnight as detailed above. The following day, the cells were subjected to the same 3HK and Ro61-8048 conditions as described above for 24 h. After incubation, 100 *μ*l of Caspase-glo 3/7 reagent (Promega, WI, USA) was added to each sample well and mixed at 500 r.p.m. on a plate shaker for 30 s followed by a 2 h incubation at room temperature. The luminescence in each well was read using the TECAN infinite M1000 plate reader at OD1 with an integration time of 1 s. Caspases 3 and 7 activity is an indicator of cell death as these proteins are only activated after apoptosis signalling events have occurred.^21^ Caspase 3 and 7 share similar substrate specificity allowing them to be assayed simultaneously.^22^

### DiOC6 assay

Non-transfected HEK293 cells and HEK-KMO(V5-6His) cells were plated out separately into 96-well plates at 5 × 10^4^ cells per well and incubated overnight at 37 °C, 5% CO_2_, 95% O_2_ in DMEM with 10% FBS, 1% l-glutamine, 1% penicillin-streptomycin. Four plates were prepared to represent each time point, i.e. 0, 3, 6 and 24 h. The next day, tissue culture medium was replaced with medium containing 0, 10, 100 or 500 *μ*M 3HK. The 3HK dose response was incubated in the presence and absence of Ro61-8048 at 10 *μ*M in concentration and, separately, in the presence and absence of Z-VAD-FMK (Promega) at 10 *μ*M in concentration. Staurosporine was applied as a positive control at a concentration of 300 nM. Cells were incubated at 37°C, 5% CO_2_, 95% O_2_ for the appropriate time, that is, 0, 3, 6 or 24 h. Following incubation, media was removed from the cells and replaced with PBS containing 100 nM DiOC6 (Thermo Fisher). DiOC6 is membrane dye which is used to detect mitochondrial membrane potential in live cells. The cells were incubated for 30 min at 37 °C, 5% CO_2_, 95% O_2_ in the dark. PBS containing DiOC6 was removed from the cells and replaced with fresh PBS. Fluorescence intensity was measured using the TECAN infinite M1000 plate reader (Tecan). The plate was read at excitation 482 nm and emission 504 nm to detect DiOC6 staining.

### 3HK Metabolism in whole cells

Non-transfected HEK293 cells and HEK-KMO(V5-His) cells were plated out separately into 96-well plates at 5 × 10^4^ cells per well and incubated overnight at 37 °C, 5% CO_2_, 95% O_2_ in DMEM with 10% FBS, 1% l-glutamine, 1% penicillin-streptomycin. The following day, the medium on the cells was replaced with 1.5 mM 3HK diluted in tissue culture medium at a volume of 100 *μ*l per well. Samples were obtained every hour from 0 to 7 h. Controls consisted of DMEM with 10% FBS, 1% l-glutamine, 1% penicillin-streptomycin and 1.5 mM 3HK incubated in the absence of cells. Each sample was precipitated in 500 *μ*l acetonitrile with 25 *μ*g/ml d5 tryptophan (internal standard for MS method) and dried down under nitrogen. Samples were re-suspended in 30 : 70 methanol:water with 0.1% formic acid and mass spectrometry analysis (as described above) was used to quantify 3HK remaining in each sample.

### Transfection of siRNA

HEK-KMO(V5-6His) cells were plated in to six-well plates at 8 × 10^5^ cells per well and incubated overnight at 37 °C, 5% CO_2_, 95% O_2_ in OPTI-MEM medium. The following day, cells were transiently transfected with siRNA for the genes corresponding to TDO, KYNU, HAAO and QPRT using Lipofectamine RNAiMAX according to the manufacturer's protocol. Each siRNA duplex was transfected both individually and simultaneously. The medium on the cells was replaced with fresh OPTI-MEM the following day. 48 h post transfection, OPTI-MEM containing 500 *μ*M 3HK was incubated with the cells for 24 h. The LDH activity assay was performed on the cell supernatants and RT-qPCR analysis was used to verify knockdown in the cells.

### RT-qPCR analysis: kynurenine pathway enzymes

Non-transfected HEK293 cells and HEK-huKMO(V5-6His) cells were collected and pelleted by centrifugation for 5 min at 1000 r.p.m. Tissue culture medium was carefully removed from the cell pellets. Total RNA was extracted from cell pellets using an RNeasy Mini kit (Qiagen, Hilden, Germany). One microgram of total RNA was used for first strand cDNA synthesis using a QuantiTect Reverse Transcription Kit (Qiagen). Expression of the genes corresponding to the kynurenine pathway enzymes were determined by real-time PCR. We used inventoried TaqMan assays and the real-time PCR assay was performed on the StepOne system (Applied Biosystems, Foster City, CA, USA). The thermal profile was 95 °C for 20 s followed by 45 cycles of 95 °C for 3 s and 60 °C for 30 s. We performed all assays as duplex reactions with an endogenous 18S RNA internal control.

### Time-lapse bright-field microscopy

HEK-KMO(V5-6His) and HEK293 cells were passaged separately on to 5 ml slide flasks (Nunc, Thermo Scientific, Leicestershire, UK) at a density of 1 × 10^5^ cells/ml. Cells of each type were incubated with medium containing either 500 *μ*M 3HK or control medium (no additives) and incubated overnight for 24 h in a warm room at 37 °C, 5% CO_2_ at maximum humidity. A Leica DMIRBE inverted microscope (Leica Microsystems) situated in the warm room was utilised to obtain images using a × 10 objective lens at 5 min intervals for 24 h. Images were captured using Leica QWIN software and composite videos exported using Quicktime player.

### Live cell imaging

HEK293 cells were plated out at 1 × 10^4^ cells/ml on poly-d-lysine-treated coverslips placed in the wells of a six-well plate in OPTI-MEM medium and incubated overnight for 16 h at 37 °C, 5% CO_2_. The following day, cells were transiently transfected with pcDNA-10His-E2-Crimson-KMO plasmid DNA using lipofectamine 2000 in OPTI-MEM medium. Transfection media was removed 6 h post transfection and replaced with DMEM with 10% FBS, 1% l-glutamine, 1% penicillin-streptomycin before incubation of the cells for a further 18 h at 37 °C, 5% CO_2_. 24 h post transfection, coverslips were transferred to the heated stage of a Zeiss LSM510 META (Zeiss, Cambridge, UK). Cells were immersed in phenol red-free OPTI-MEM medium containing 5 mM CaCl_2_, Annexin-V-FLUOS (Roche) at 1 : 100 dilution (diluted from the ready-to-use stock) and 500 *μ*M 3HK and incubated at 37 °C, 5% CO_2_ to allow time-lapse images to be acquired. Images were obtained using a Plan-Apochromat 63x NA1.4 oil lens with no zoom (resulting pixel size 147 nm × 147 nm). For visualisation of 10His-E2-Crimson-KMO-expressing cells, the fluorescent E2-Crimson protein was excited at 611 nm and the emitted light recorded at 646 nm. For visualisation of apoptotic cells, the Annexin V fluorophore was excited at 488 nm and the emission was recorded using a narrow emission filter BP 515-565 nm. Cells were incubated for 24 h with a time-lapse of 3 min between image acquisition. Data was collected using the corresponding LSM 5 software program (Zeiss, Oberkochen, Germany).

### Statistical analysis

Differences between controls and experimental samples were analysed using an unpaired Student's *t*-test using GraphPad Prism software (GraphPad Software Inc., CA, USA).

## Figures and Tables

**Figure 1:**
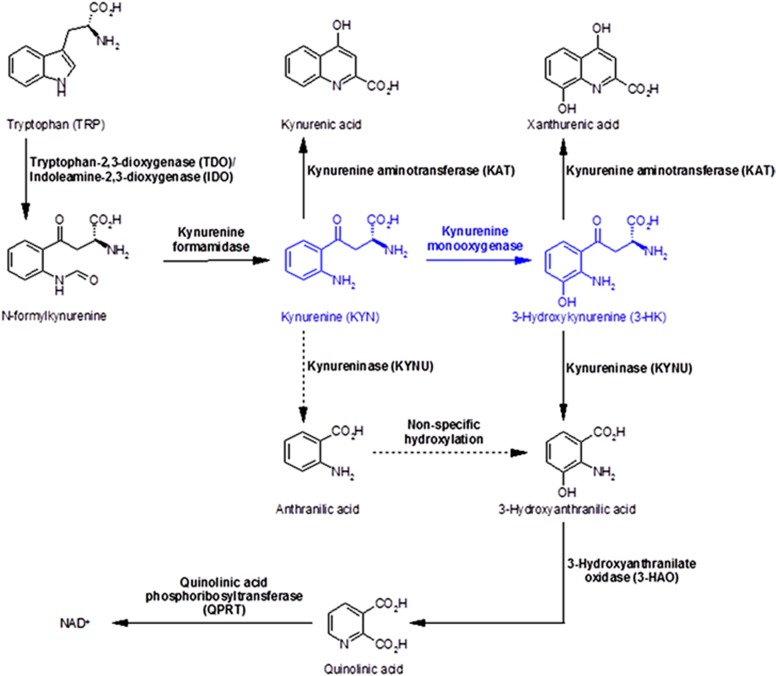
The kynurenine pathway of tryptophan metabolism with the KMO catalytic reaction indicated in blue

## Abbreviations

KMO: Kynurenine 3-monooxygenase
3HK: 3-hydroxykynurenine
iNOS: inducible nitric oxide synthase
TRP: tryptophan
KYN: kynurenine
TDO: tryptophan-2,3-dioxygenase
IDO: indoleamine-2,3-dioxygenase
KAT1: kynurenine aminotransferase 1
KAT2: kynurenine aminotransferase 2
KYNA: kynurenic acid
GABA: *γ*-Aminobutyric acid
3HAA: 3-hydroxyanthranilic acid
3HAO: 3-hydroxyanthranilic acid oxidase
QUIN: quinolinic acid
NAD: nicotinamide adenine dinucleotide
HEK: human embryonic kidney cells
NMDA: N-methyl-D-aspartate
NADPH: Nicotinamide adenine dinucleotide phosphate
QPRT: quinolinate phosphoribosyl transferase
HAAO: 3-hydroxyanthranilate 3, 4-dioxygenase
KAT3: kynurenine aminotransferase 3
CHO: Chinese hamster ovarian cells
KP: kynurenine pathway
NAD: nicotinamide adenine dinucleotide
LC-MS: liquid chromatography-mass spectrometry
LDH: lactase dehydrogenase
DiOC6: 3,3′-dihexyloxacarbocyanine iodide
HCS: high content screening
